# Distinct Mechanisms Underlying Tolerance to Intermittent and Constant Hypoxia in *Drosophila melanogaster*

**DOI:** 10.1371/journal.pone.0005371

**Published:** 2009-04-29

**Authors:** Priti Azad, Dan Zhou, Erilynn Russo, Gabriel G. Haddad

**Affiliations:** 1 Department of Pediatrics, Section of Respiratory Medicine, University of California San Diego, La Jolla, California, United States of America; 2 Department of Neuroscience, University of California San Diego, La Jolla, California, United States of America; 3 The Rady Children's Hospital, San Diego, California, United States of America; Leiden University Medical Center, Netherlands

## Abstract

**Background:**

Constant hypoxia (CH) and intermittent hypoxia (IH) occur during several pathological conditions such as asthma and obstructive sleep apnea. Our research is focused on understanding the molecular mechanisms that lead to injury or adaptation to hypoxic stress using *Drosophila* as a model system. Our current genome-wide study is designed to investigate gene expression changes and identify protective mechanism(s) in *D. melanogaster* after exposure to severe (1% O_2_) intermittent or constant hypoxia.

**Methodology/Principal Findings:**

Our microarray analysis has identified multiple gene families that are up- or down-regulated in response to acute CH or IH. We observed distinct responses to IH and CH in gene expression that varied in the number of genes and type of gene families. We then studied the role of candidate genes (up-or down-regulated) in hypoxia tolerance (adult survival) for longer periods (CH-7 days, IH-10 days) under severe CH or IH. Heat shock proteins up-regulation (specifically Hsp23 and Hsp70) led to a significant increase in adult survival (as compared to controls) of P-element lines during CH. In contrast, during IH treatment the up-regulation of Mdr49 and l(2)08717 genes (P-element lines) provided survival advantage over controls. This suggests that the increased transcript levels following treatment with either paradigm play an important role in tolerance to severe hypoxia. Furthermore, by over-expressing Hsp70 in specific tissues, we found that up-regulation of Hsp70 in heart and brain play critical role in tolerance to CH in flies.

**Conclusions/Significance:**

We observed that the gene expression response to IH or CH is specific and paradigm-dependent. We have identified several genes Hsp23, Hsp70, CG1600, l(2)08717 and Mdr49 that play an important role in hypoxia tolerance whether it is in CH or IH. These data provide further clues about the mechanisms by which IH or CH lead to cell injury and morbidity or adaptation and survival.

## Introduction

Constant or intermittent hypoxia frequently occurs in disease states. For example, intermittent hypoxia (IH) is associated with obstructive sleep apnea, central hypoventilation syndrome and intermittent vascular occlusion in sickle cell anemia. Constant hypoxia (CH) is associated with pulmonary disease such as asthma, and congenital heart disease with right to left shunt. Hypoxia can occur even under normal conditions such as at high altitude. Whether for IH or CH, various studies, using rodents as animal models have examined experimentally the effects of hypoxia on specific tissues such as heart, brain, and kidneys [1], [2], [3], [4], [5]. These studies have demonstrated that the response to low O_2_ is not only dependent on intensity and duration of the stimulus but also on the paradigm used. For example, CH and IH are very different in their effect on growth, proliferation, generation of reactive O_2_ species, and neuronal injury [2], [6], [7], [8], [9]. Furthermore, *in vivo* studies have shown organ-specific phenotypic differences to low O_2_ such as hypertrophy in heart or decrease in myelination and NAA/Cr ratios in brain [5], [6], [7].

The differences in the fundamental mechanisms underlying the responses to IH and CH are however not well understood. In spite of the fact that we know that IH and CH involve a differential expression of genes and pathways [5], [10], [11], we do not have a good appreciation as to whether these genes are important for the observed phenotype. While it is possible after obtaining results from microarray data to study the role of single or multiple genes in inducing the phenotype, it is rather difficult to perform such studies rather quickly *in vivo* in mice. Another way to approach this problem is to study some of these questions in a model organism, as we have done in the past [12], [13], [14], prove the role of certain genes in the phenotype and then investigate orthologs in mammals, such as rodents, and ultimately in humans. The advantage of using model systems such as *Drosophila melanogaster* is the relative speed with which one can perform such studies, especially that a) more than 65–70% of human disease genes are present in *Drosophila* and b) this model has served well not only the discovery of the relation of such genes to diseases [15] but also in the understanding of how such genes induce the disease itself [16].

Our previous studies have shown that *Drosophila* is extremely resistant to hypoxia or even anoxia for a few hours [12], [13], [17]. The brain of these animals, for example, does not suffer from any damage (by light or electron-microscopy) after a period of anoxia that can induce irreversible injury and death in rodents [13], [17], [18]. In addition, the *Drosophila* model system offers other advantages such as a short life span, a large progeny size and the availability of genetic markers and tools. In the past, we have used similar approaches including forward and reverse genetic approaches, as in this current work, and have identified several hypoxia-regulated genes [14], [18]. We have also done microarray studies that have provided us with insight regarding tolerance of flies to long term (over many generations) hypoxia [14]. In this study, we focus on gene expression changes associated with severe short term constant (CH) or intermittent hypoxia (IH) in adult flies. Our hypothesis is that CH induces a different gene expression profile than IH in *Drosophila* and that these expression changes play an important role in inducing tolerance and protecting the organism against hypoxic injury.

## Results

### Gene expression changes during IH and CH

**Figure 1:**
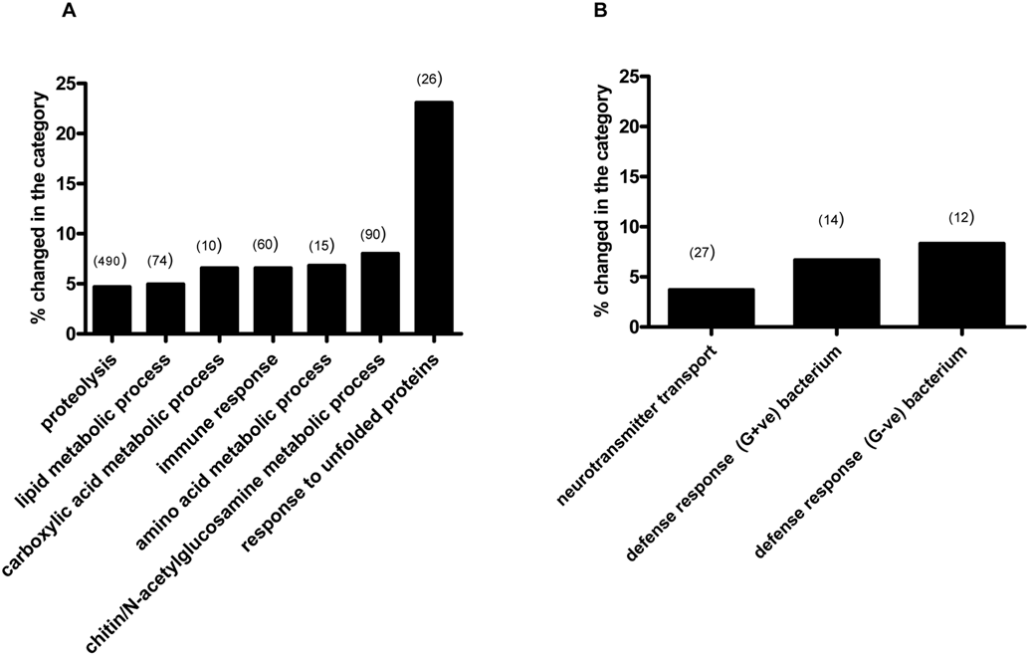
Over-expressed genes in various biological processes during hypoxia (CH or IH). (A): Over-expressed genes in biological processes during constant hypoxia. (B): Over-expressed genes in biological processes during intermittent hypoxia. The bars represent over-expressed biological processes in terms of percentage of genes changed in the category as calculated by GENMAPP as shown in table 1. The number in parentheses represents the number of genes measured as shown in Table 1.

**Figure 2:**
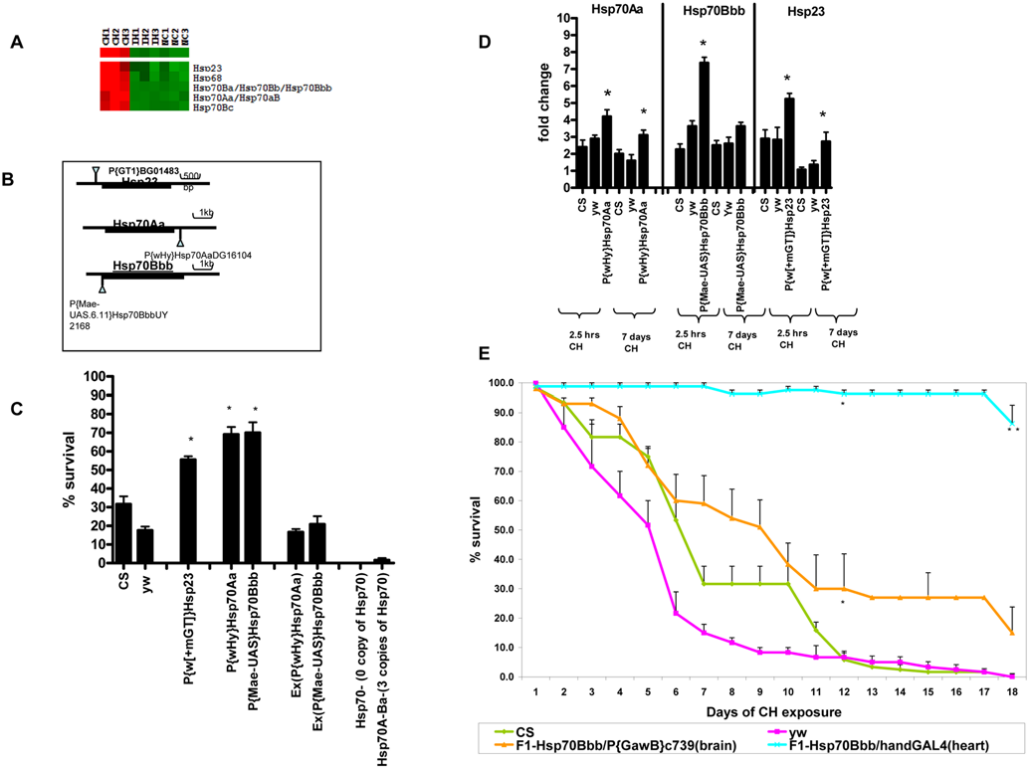
Role of Hsp70 and Hsp23 during CH. (A): The microarray results were clustered using GenMapp and viewed with Treeview. Upregulated genes are shown in red and downregulated genes are shown in green. Heat shock proteins specifically Hsp23, Hsp68 and Hsp70, are significantly over-expressed during constant hypoxia (CH1, CH2 and CH3) as compared to normoxia controls (NC1, NC2 and NC3) and intermittent hypoxia replicates (IH1, IH2 and IH3). (B): Genomic location of P-element for genes Hsp23, Hsp70Aa and Hsp70Bbb. (C): Functional testing of Hsp23 and Hsp70 genes during CH. Percent survival of adult flies after exposure to 1.5% O_2_ CH for 7 days. CS and yw serve as controls. Hsp70 and Hsp23 P-element lines showed significantly higher survival than CS, yw controls (P<0.05). In contrast, P-element excision lines (Ex) and Hsp70- lines showed survival similar to or less than controls. Each bar represents the average of at least three tests for each line and error bars represent the standard errors. * with unpaired t-test P<0.05. (D): Real-time PCR analysis to validate upregulation of Hsp70Aa, Hsp70Bbb and Hsp23 in controls and P-element lines after exposure to CH. Hsp-P-element lines showed significantly higher mRNA levels of heat shock proteins than controls after 2.5 hours and 7 days of CH exposure(P<0.05). * With unpaired t-test comparing the fold change of P-element lines with yw normoxia control, P<0.05. (E): Testing for survival of adult F1 flies expressing Hsp70 in various tissues using the GAL4 drivers at 1.5% O_2_ CH. The graph represents the percent survival of CS, yw and the F1 progeny obtained by crossing UAS-Hsp70Bbb with Hand(Heart) and c736(Brain)-GAL4 drivers. Over-expression of Hsp70 in cardioblasts, pericardial cells and hemocytes(Hand) leads to remarkable survival as compared to controls (at day 18, P<0.0001). * with unpaired t-test P<0.05. ** P<0.0001.

**Figure 3:**
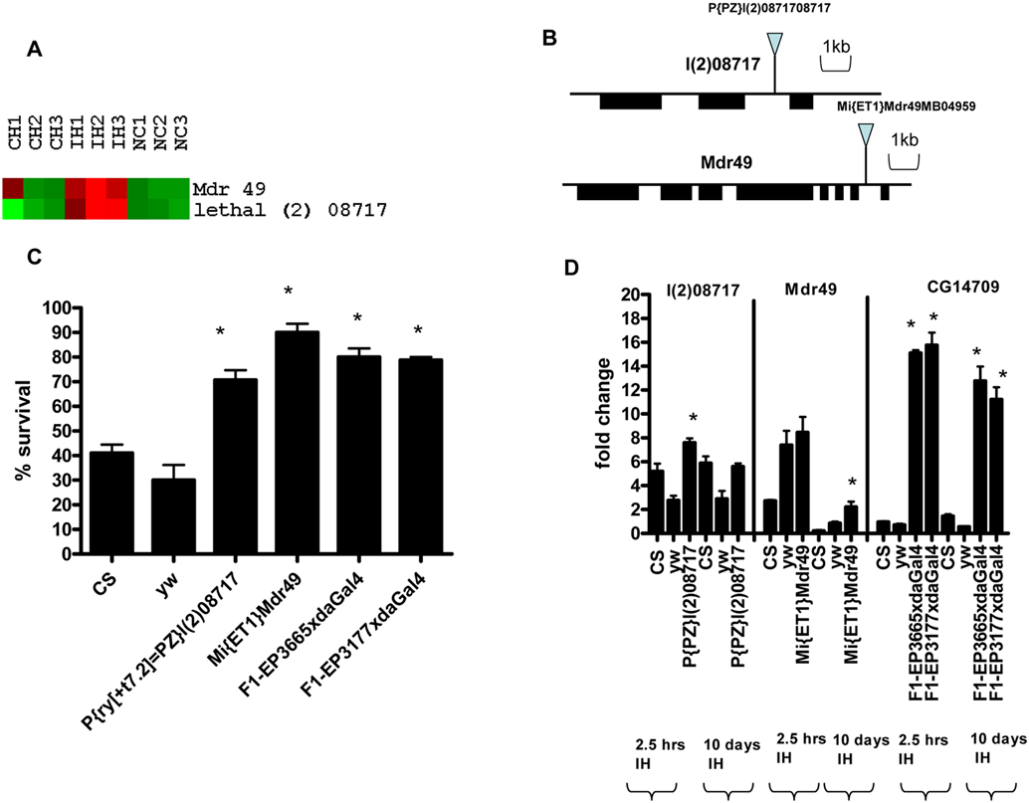
Role of Mdr49 and l(2)08717 genes during IH. (A):Over-expression of Mdr 49 and l(2)08717 during intermittent hypoxia as demonstrated by microarray analysis. Upregulated genes are shown in red and downregulated genes are shown in green. IH1, IH2 and IH3 represent replicates exposed to intermittent hypoxia for 2.5 hours. Mdr49 and l(2)08717 are specifically upregulated during IH. NC1, NC2 and NC3 represent the normoxia controls and CH1, CH2 and CH3 represent replicates exposed to constant hypoxia for 2.5 hours. (B): Genomic location of P-element for genes l(2)08717 and Mdr49. (C): Functional testing of l(2)08717, Mdr49 and CG14709 genes during IH. Percent survival of adult flies after exposure to 21%–1.5% O_2_ IH for 10 days. CS and yw serve as controls. P{PZ}l(2)08717 and Mi{ET1}Mdr49 lines showed significantly higher survival as compared to controls(CS, yw, P<0.05). Each bar represents the average of at least three tests for each line and error bars represent the standard errors. * With unpaired student t-test comparing the % survival of P-element lines with CS and yw controls, P<0.05. (D): Real-time PCR analysis to validate upregulation of l(2)08717, Mdr49 and CG14709 genes after exposure to IH in controls and P-element lines. After normalizing the values with actin, fold change was calculated in comparison to relative mRNA levels at normoxia. For l(2)08717, Mdr49 and CG14709 genes relative mRNA levels and fold change was measured in CS, yw and the P-element lines after 2.5 hours and 10 days. P-element lines of Mdr49 and CG14709 showed increased expression after 2.5 hours and 10 days as compared to controls, P<0.05. * With unpaired t-test comparing the fold change of P-element lines with yw normoxia control, P<0.05.

**Figure 4:**
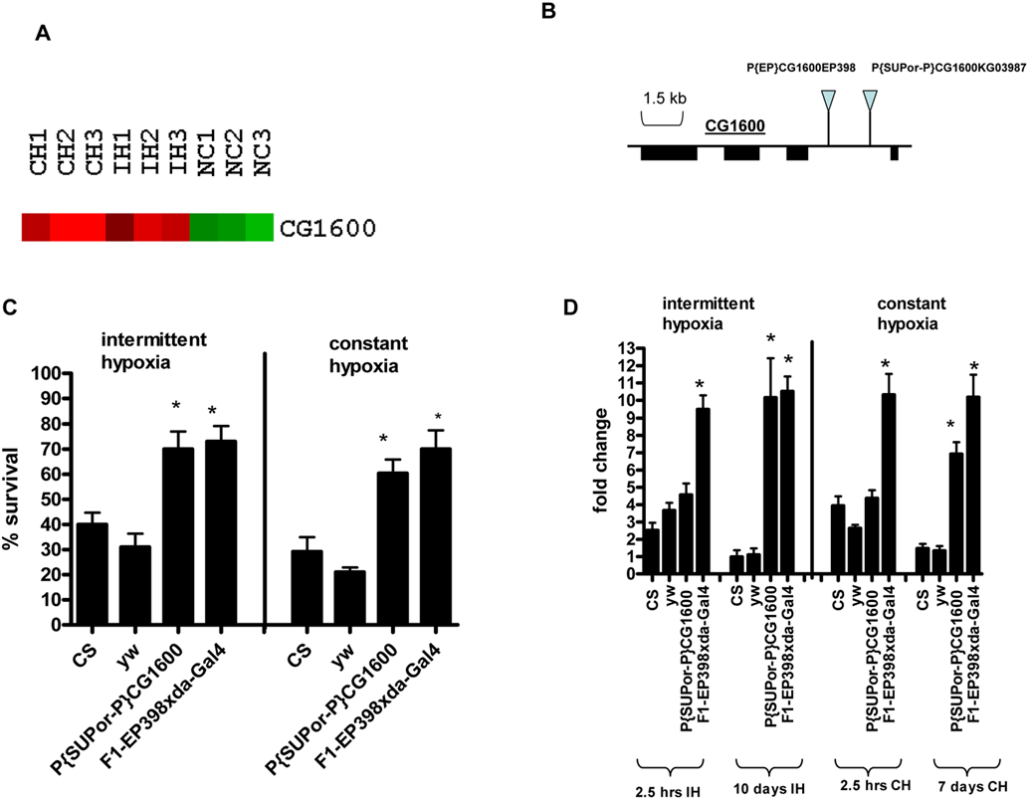
CG1600 gene has an important role under both paradigms- CH and IH. (A): Over-expression of CG1600 during IH as well as CH as demonstrated by microarray analysis. Upregulated genes are shown in red and downregulated genes are shown in green. IH1, IH2 and IH3 represent replicates exposed to intermittent hypoxia for 2.5 hours. NC1, NC2 and NC3 represent the normoxia controls and CH1, CH2 and CH3 represent replicates exposed to constant hypoxia for 2.5 hours. (B): Genomic location of P-elements for gene CG1600. (C): Functional testing of CG1600 gene during CH and IH. Percent survival of adults flies after exposure to 21%–1.5% O_2_ IH for 10 days. Percent survival of adult flies after exposure to 1.5% O_2_ CH for 7 days CS and yw serve as controls. P-element lines of CG1600 survived much better than controls under both CH and IH (P<0.05). Each bar represents the average of at least three tests for each line and error bars represent the standard errors.* with unpaired t- test P<0.05. (D): Real-time PCR analysis to validate upregulation of CG1600 gene in controls and P-element lines after exposure to IH and CH. After normalizing the values with actin, fold change was calculated in comparison to relative mRNA levels at normoxia. For CG1600 gene relative mRNA levels and fold change was measured in CS, yw and the F1 progeny of EP line x dal-Gal4 and P-element line after 2.5 hours and 10 days for IH and CS, yw and the P-element after 2.5 hours and 7 days for CH. P-element lines of CG1600 genes showed several fold higher expression than controls even after 7 days CH or 10 days IH. * With unpaired t-test comparing the fold change of P-element lines with yw normoxia control, P<0.05.

The microarray results showed that there were many fewer significantly altered genes following IH (12 up-regulated and 4 down-regulated genes) (Table S1) as compared to CH (94 up-regulated and 70 down-regulated genes) (Table S2). Using MAPPFinder in conjunction with GENMAPP, we discovered that there were several gene families that were over-represented in CH treated flies, such as those involved in the response to unfolded proteins, chitin, lipid, carboxylic acid, amino acid-metabolic processes, and the immune response (Table 1, Figure 1A). Indeed, the heat shock protein family was the most up-regulated group in CH and this was exclusive to this treatment (Z score = 6.7; Table 1, S2) (Figure 1, 2A). In contrast, during IH biological processes primarily involved in neurotransmitter transport and defense response were over-represented (Figure 1B, Table 1). Indeed, multidrug resistance proteins (Mdr 49, 50) were up-regulated and were exclusively altered in IH (Z score = 26.82, Table 1, S1) (Figure 1B, 3A). There were however genes such as CG3384 and CG1600 that were upregulated in both IH and CH conditions (Table S1, S2). These genes are involved in chitin metabolic processes and oxidoreductase activity, respectively. We validated the expression profiles of candidate genes (Hsp23, Hsp70, CG1600, Mdr49, l(2)08717 by real-time PCR and found these data to be consistent to that observed in microarrays (Figure 2D,3D, 4D).

**Table 1:** Over-represented biological processes after 2.5 hours of CH (1% O_2_) or IH (1%–21% O_2_) exposure as computed by GENMAPP.

GOID	Constant Hypoxia (CH)	# Changed	# Measured	# in GO	% Changed	% Present	Z Score	PermuteP
	GO Name							
6986	response to unfolded protein	6	26	31	23.077	83.871	6.766	0
19752	carboxylic acid metabolic process	1	10	10	6.566	98.020	3.74	0.001
6508	proteolysis	24	490	532	4.704	93.031	3.541	0.003
6520	amino acid metabolic process	1	15	16	6.818	97.778	3.231	0.007
6030	chitin metabolic process	7	90	99	8.000	91.743	3.572	0.008
7219	Notch signaling pathway	5	40	41	11.364	97.778	3.801	0.008
6950	response to stress	3	19	19	6.135	95.882	3.03	0.012
48102	autophagic cell death	5	61	62	8.824	98.551	3.379	0.013
6967	+ regulation of antifungal peptide biosynthetic process	2	7	7	28.571	100.000	4.443	0.018
19642	anaerobic glycolysis	1	1	1	100.000	100.000	6.274	0.021
6629	lipid metabolic process	6	74	79	4.975	97.573	2.303	0.038
6952	defense response	1	27	29	6.579	87.356	2.309	0.04
6955	immune response	5	60	66	6.579	91.566	2.309	0.05

GOID	Intermittent Hypoxia (IH)	# Changed	# Measured	# in GO	% Changed	% Present	Z Score	PermuteP
	GO Name							
6855	multidrug transport	1	1	1	100.000	100.000	26.82	0.002
6810	transport	5	704	735	0.431	95.868	2.855	0.017
50830	defense response to Gram-positive bacterium	1	14	15	6.667	93.750	6.795	0.022
50829	defense response to Gram-negative bacterium	1	12	14	8.333	85.714	7.628	0.023
6961	antibacterial humoral response	1	16	19	6.250	84.211	6.57	0.027
6836	neurotransmitter transport	1	27	28	3.704	96.429	4.982	0.049

To determine which altered genes has a functional role and is crucial in sustaining survival under severe hypoxia, we used P-element lines. We first focused on the genes which had single P-element insertions within or around the genes of interest (i.e., altered in the microarrays). We argued then that testing the survival of such P-element (or EP lines) in either IH or CH will allow us to gain insight into the role of these genes. We therefore tested the survival of these fly lines during 1.5% O_2_ along with CS and yw serving as controls (see Methods).

### Gene regulation during CH: Role of Hsp70 and Hsp23

Seventeen percent of the total P-element lines that were tested during CH showed a role for the up-or down-regulated genes. Among the upregulated genes, heat shock proteins and specifically Hsp70 and Hsp23, seem to play an important role in hypoxia tolerance. The survival rate was 55% for Hsp23 and ∼70% for the Hsp70Aa and Hsp70Bbb P-elements as compared to control (31%) (P<0.05, Figure 2C, Table S3). In addition, when the Hsp70 P-elements were precisely excised, their increased survival was eliminated (Fig. 2C). Interestingly, when we used fly lines that had no copies of Hsp70 or even half of the copies for Hsp70Bb, Bbb, and Bc expressed, adult flies had markedly reduced survival and even lower survival than controls (Figure 2C). Furthermore, by analyzing the gene expression profiles (real-time PCR) in the P-element lines and controls during hypoxia (2.5 hrs or 7 days), we found that P-element lines had much higher expression levels of heat shock proteins than controls (Figure 2D) such as five fold higher mRNA level of Hsp70Bbb as compared to controls after 2.5 hours (P<0.05, Figure 2D). The P-element lines Hsp70Aa and Hsp23 also had two fold higher expression levels in their mRNA than controls after 2.5 hours as well as after 7 days of CH (P<0.05, Figure 2D).

In order to further dissect the mechanisms of protection as it pertains to heat shock 70, we over-expressed Hsp70 in various tissues such as heart, muscles and brain utilizing progenies of crosses with specific GAL4 drivers. We then subjected the F1 progeny of such crosses to severe hypoxic stress by exposing them to 1.5% O_2_ CH and assessing adult survival along with controls. By manipulating the spatial expression of Hsp70 *in vivo*, we made several interesting observations. Ubiquitous expression of Hsp70 (using da-Gal4) causes lethality at the larval stage. We also observed that expressing Hsp70 in the heart (specifically cardioblasts, pericardial cells and lymph gland-which is a hematopoietic organ in flies; [19], [20]) increased survival of the adult flies remarkably (Figure 2E). After 12 days of exposure to 1.5% O_2_ CH, the F1-UAS-Hsp70/HandGal4 progeny continued to have almost full survival (∼97% as compared to controls which had 6% survival, P<0.0001; Figure 2E). Furthermore, even after 19 days of CH exposure, at which point there were no controls alive, F1-UAS-Hsp70/HandGal4 progeny had still 86% adult survival. Over-expressing Hsp70 in brain (F1-UAS-Hsp70/P{GawB}c739- specific expression in mushroom body and some parts of antennal lobes [21]) also induced a better survival than controls (P = 0.017, Figure 2E). In contrast, over-expressing Hsp70 exclusively in the muscles (F1-UAS-Hsp70/P{GawB}DJ667), glial cells (F1-UAS-Hsp70/Eaat1) and nervous system (F1-UAS-Hsp70/elav-GAL4) did not seem to have any beneficial effect on adult survival under hypoxia (P>0.05, t-test). The fly stocks of GAL4 drivers alone (without Hsp70 over-expression) did not survive better than controls (P>0.05, t-test). It is interesting to note that over-expressing Hsp70 in the entire nervous system (neurons-CNS and PNS) (elav) had a deleterious effect on adult survival. In contrast, over-expressing Hsp70 only in specific parts of brain (alpha and beta lobe Kenyon cells (intrinsic neurons) of the Mushroom bodies) provides survival advantage over controls.

### Gene regulation during IH: Role of Anion/Cation symporter and multidrug resistance genes

While screening the P-element lines for adult survival during 1.5% O_2_ IH (Table S4) we found that P{PZ}l(2)08717 had a much higher survival (70% survival) than controls (CS-41% and yw-30% survival, P<0.05; Figure 3C). In fact this P-element line showed around five fold higher level of mRNA than that of yw (control) after 2.5 hours exposure of IH (Figure 3D). After 10 days of IH, the mRNA levels P{PZ}l(2)08717 was still 2.5 fold higher than yw (P<0.05, Figure 3D). Another family that was predominantly and exclusively upregulated in IH was the Multi-drug resistance proteins (Figure 3A). The P-element line Mi{ET1}Mdr49 showed more than double (∼90%) adult survival in IH as compared to controls (∼40%) after 10 days of exposure (P<0.05,Figure 3C). Real-time PCR results showed that Mi{ET1}Mdr49 has about two fold higher Mdr49 expression than controls even after 10 days of IH exposure (P<0.05, t-test, Figure 3D), suggesting that sustained increased expression of Mdr49 is beneficial in survival over longer periods of IH treatment. In order to reinforce our hypothesis that Multi-drug resistance/transport is linked to IH tolerance, we tested CG14709 gene which belongs to the same Multi-drug resistance protein family [22]. We over-expressed this gene ubiquitously using da-Gal4 driver and confirmed that the F1 progeny had >10 fold higher mRNA level than controls (Figure 3D). The increased expression of this gene provided marked adult survival to the F1 progeny of the EP lines (EP3655 and EP3177) during IH exposure as shown by a ∼70% survival of these lines as compared to the controls (∼40% survival) (P<0.05, Figure 3C).

### Gene Regulation during IH and CH: CG1600 gene and Role of its Oxidoreductase activity

CG1600 gene is up-regulated in both CH and IH. It is involved in zinc binding and has oxidoreductase activity. When over-expressed, it led to better survival of flies under both treatments (Figure 4A–C). The F1 progeny of EP398 line and P{SUP}CG1600 line showed almost double (∼70%) percent survival during both IH and CH than in controls (P<0.05; Figure 4C). The real-time PCR data demonstrated significantly higher expression of CG1600 gene in the P-element (P-SUP) and EP398(F1 progeny) line under both regimes-CH and IH after 7 and 10 days respectively (P<0.05;Figure 4D). This suggests that the increased expression (>4fold) of CG1600 gene in these P-element lines leads to their increased survival (∼double) under both IH and CH regimes as shown above.

## Discussion

The mechanisms underlying injury from hypoxia or adaptation to this stress are not well delineated, although previous studies have shed some light on certain fundamental aspects of the response to hypoxia [23], [24], [25], [26], [27], [28]. In this study, we employed gene expression analysis coupled with the functional studies of the role of specific genes in survival to IH or CH *in vivo* using *Drosophila* as a model system and made two major observations. First, we found that gene expression was dependent on the hypoxia paradigm, such that IH induced genes predominantly involved in transport and defense, whereas CH strongly induced genes involved in stress response and metabolic processes. Furthermore, based on survival and gene expression data, it appeared that Mdr49 and l(2)08717 genes played an important role in IH. In contrast, the survival advantage in CH was provided specifically by heat shock proteins Hsp70 and Hsp23. Second, it appeared that the role of the genes induced under CH or IH was not generalized but very much specific to the paradigm. For example, Hsp70, which played an important role in CH tolerance, did not do so when over-expressed and tested in IH. Similarly P-element lines which over-expressed Mdr49 and l(2)08717 genes showed survival in IH but did not show a survival advantage over controls in CH.

In order to further appreciate the functional role of the specific candidate genes, we performed experiments in which we used the UAS-Gal4 technique to differentially express such genes in specific tissues. For example, we have shown that the specific over-expression of Hsp70 in heart (cardial cells, pericardial cells and hemocytes) and to a certain degree in brain (mushroom body and antennal lobes) of flies increased survival in CH. This survival in CH did not occur when Hsp70 was over expressed in other tissues such as in muscles. This differential survival caused by tissue specific Hsp70 over-expression highlights the importance of specific tissues in survival during hypoxic stress. Our observation that ubiquitous expression of Hsp70 caused lethality at the larval stage in flies could be dependant on the level (amount) of over-expression of Hsp70. Although mechanisms and signaling pathways for protection conferred by Hsp70 remain to be elucidated, it is known that Hsp70 has diverse functions, such as acting as a chaperone as well as in apoptosis [29], through the interaction with c-jun amino terminal kinase (JNK), BCL-2 Interacting Domain (Bid) and BCL2-associated athanogene (BAG1) [30], [31]. Either of these mechanisms may be playing a role in survival in our flies when subjected to CH and when Hsp70 is over-expressed. It is interesting to note in this regard that previous studies have shown that the heat shock family is induced and is important in stress such as hypoxia but these studies had not appreciated the differences between IH and CH and their gene expression underpinnings or the specificity of tissue importance for the survival of the whole organism [32], [33], [34], [35], [36]. This demonstrates the utility of *Drosophila*, once again as a model organism to further dissect the mechanism(s) involved in hypoxia tolerance.

We have shown in this work that two single genes- Mdr49 and l(2)08717 play an important role in terms of adult survival in IH. l(2)08717 gene is a high affinity inorganic phosphate: sodium symporter and belongs to the Anion/Cation Symporter(ACS) family of transporters. Interestingly, l(2)08717 gene has protein homology with human sailin protein which is associated with lysosomal storage diseases such as Salla disease (SD) and Infantile sialic acid storage disease (ISSD) [37]. Hypoxia-induced upregulation of sialin has been reported in an *in-vitro* study using cultured cancer cells [38]. At this point, it is not clear as to how this gene confers tolerance to IH. In humans sialin is thought to play a significant role in regulating lysosomal pH, through an anion conductance or coupled movement of protons [39]. Whether l(2)08717 symporter activity maintains lysosomal pH and leads to better survival during IH is not known at this time, but previous studies in flies have shown that gene mutations linked with lysosomal trafficking pathways lead to synaptic dysfunction, neuronal degeneration and decrease in lifespan in adults [40], [41]. Furthermore, *in vitro* studies have shown that l(2)08717 gene is a putative target of Hairy and Clock genes in *Drosophila* which are major regulators of hypoxia tolerance, circadian rhythms and a variety of metabolic pathways suggesting other potential mechanism(s) by which this gene leads to IH tolerance [42], [43].

Our finding regarding the role of Mdr49 gene exclusively during IH is also intriguing. The Mdr family is induced in multi-drug resistance cell lines which are resistant to a broad spectrum of compounds, including the ones used for cancer chemotherapy [44]. Although the mechanism(s) by which Mdr49 leads to IH tolerance is not known at this time, we have previously identified Mdr49 as one of the putative targets of *Drosophila* ADAR gene by using immunoaffinity enrichment of inosine-containing mRNA, DNA microarrays and sequence comparison data [45]. Adenosine deaminase acting on RNA works through RNA editing and alters protein structure and function. For example, in the mammalian brain, its activity results in changes in the functional properties of neurotransmitter receptors such as glutamate and serotonin as well as channels and transporters [46]. We have previously shown that in dADAR−/− flies become hypoxia-sensitive and have premature neurodegeneration [45]. We have also shown that ADAR plays a regulatory in ROS metabolism [47], which can also be important in IH.

In summary, this study is the first that we know of that has examined the gene expression changes and the role of specific genes involved in tolerance to both IH and CH using *Drosophila* as the model system. This study has demonstrated that a) gene expression profiles are specific to the stimulus paradigm, b) specific genes play an important role in protecting *Drosophila* and promoting survival in severe stress, and c) gene expression during IH or CH in specific tissues enhances survival of the whole fly.

## Methods

### *Drosophila* stocks

Wild type Canton S (CS) stock was obtained from *Drosophila* Stock center (Bloomington, Indiana, USA) and used for studying changes in gene expression using microarrays. The P-element insertion stocks of the genes that were up- or down-regulated by hypoxia treatments (IH and CH) and the tissue-specific GAL4 drivers were obtained from *Drosophila* Stock centers (Bloomington, Indiana, USA, and Szeged, Hungary). Hsp70-(no copies of Hsp70) and Hsp70A-Ba-(3 copies of Hsp70) fly stocks were generous gift from Dr. Brian Bettencourt's Lab (University of Massachussets Lowell, MA, USA). Hand-Gal4 driver was generously provided by Dr. Rolf Bodmer's Lab (Burnham Institute, CA, USA). Flies were maintained on standard-cornmeal *Drosophila* medium in an incubator at temperature of 25°C and 30–50% humidity.

### Hypoxia treatments (CH, IH and NC)

In order to determine the hypoxia level for fly exposure, we performed a pilot study based on behavioral changes and mortality. At 1% CH or IH adult flies showed very slight movement, did not lay eggs and started to die after 3 days. We chose a time period of 2.5 hours to eliminate the effect of starvation based on a preliminary study where we examined the effect of starvation, hypoxia and a combination of both (unpublished observations). Flies were exposed in specially designed computerized chambers which can modulate the level of O_2_ using a combination of Oxygen (O2) and Nitrogen (N2) with Oxycycler hydraulic system (Model A44x0, BioSpherix, Redfield, NY) and ANA-Win2 Software (Version 2.4.17, Watlow Anafaze, CA). Twenty-five CS males and females (5–6 days old adult) were exposed to 2.5 hours of 1% O_2_ CH or 1–21% O_2_ IH treatments. For CH, the O_2_ level was maintained at 1% O_2_ continuously. For IH, the cycle consisted of a 4 min period of 1% O_2_ concentration alternating with a 4 min of 21% O_2_ concentration. The ramp time was 1 min for 1%–21% O_2_ and around 10 minutes for 21%–1%O_2_. Hence, the total time of one complete IH cycle is ∼20 min. Temperature and humidity were monitored and maintained at 22–24°C and 30–50% respectively. As a control, 25 males and females (5–6 days old adult) CS were exposed to 2.5 hours of normoxia (NC) and were kept in the same room and exposed to the same level of light and noise.

### Microarrays and Data Analysis

In this study, GeneChip® Drosophila Genome 2.0 Array (Affymetrix, Santa Clara, CA) were used. Three arrays were used per treatment of CH, IH and NC. Total RNA was extracted from each sample using Trizol (Invitrogen, Carlsbad, CA) followed by clean up with RNeasy Kit (Qiagen, Carlsbad, CA). All of the steps of processing the RNA, hybridization to the Affymetrix GeneChips®, washing and scanning were done according to protocols recommended by Affymetrix using an Affymetrix GeneChip fluidic station and scanner (Affymetrix, Santa Clara, CA). The raw data were normalized using Bioconductor Affy software (www.bioconductor.org/packages/2.0/bioc/html/ affy.html) and the normalized data for each spot from the arrays were analyzed for statistical significance using Web-based VAMPIRE microarray suite [48]. A spot was found differentially expressed between two samples when the threshold of false discovery rate (fdr) was smaller than 0.05. Fold changes of 1.5-fold (for up-regulated genes) and 0.67-fold (for down-regulated genes) were considered as significance limits. Only if the average of all three replicates had value >1.5 fold and all the replicates changed in the same direction (up or down regulated) did we consider such genes to be significantly regulated by hypoxia. Using MAPPFinder in conjunction with GENMAPP, we computed the P and Z scores of the over-expressed processes in individual GO categories [49]. The microarray analysis data can be retrieved using access number GSE14981 in the Gene Expression Ominibus database at http://www.ncbi.nlm.nih.gov/geo.

### Quantitative Real-time PCR analysis

Total RNA was extracted from flies (CS, yw and P-elements) under normoxia, CH or IH for 2.5 hours, 7 days (CH) or 10 days (IH) using Trizol (Invitrogen, Carlsbad, CA). cDNA was produced from total RNA through RT-PCR using Superscript III First-Strand Synthesis system (Invitrogen, Carlsbad, CA). Real-time PCR was performed using a GeneAmp 7500 sequence detection system using POWER SYBR Green chemistry (Applied Biosystems, Foster City, CA). Primers used were Hsp70Bbb (fwd: GCGCTTAAAAGCACGAGTTG; rev: CGGTTCCATTTTGTAATCCGTA); Hsp70Aa (fwd: GCGGTAGGTCATTTGTTTGG; rev:CGAAGCAACGAGAACAGTGC), Hsp23(fwd: TGTGAAGGAGAATCCCAAGG ; rev: ATAATGCAGGGCATCTCTCG); l(2)08717(fwd:TTCTTTGGCTACATCG- TGACC; rev: CAATCCGTACATCAGCATGG); CG1600 (fwd: CATCGCCTCCTATCACTTTGCC;rev:CTCGTACAGGCATTCGTTCC);Mdr49(fwd:AAAGCTCTTCCTGGAGGCAATG; rev: GATCGGGAAGATCACGCTCGTC); CG14709 (fwd: GCTGAGTAGAACCGCTTTGG, rev: AGCAGATTGACCACCTGACC)

The expression level of Actin was used to normalize the results (fwd: CTAACCTCGCCCTCTCCTCT; rev: GCAGCCAAGTGTGAGTGTGT). The array data of this study as well as our previous study [14] shows that actin did not change by short term (IH or CH) or long-term (CH-over many generations) hypoxia exposure in flies. The fold change was calculated using expression level of yw in normoxia which was used as control for all the P-element lines.

### Functional assays

#### a) Survival of adult P-elements lines in IH and CH

Five vials each containing ten adult males and females (3–5 days old, n = 100) were used for testing survival of the P-element lines and controls at 1.5% O_2_ (continuously 24 hours a day) CH for 7 days. We chose 1.5% O_2_ for longer durations due to technical feasibility (flies can move around at this level of O_2_ and hence donot get stuck in the food). The rationale for using 7 days was based on a pilot study which was done with the controls CS and yw. It was observed that controls had a mortality of 50% or less (which allowed us to distinguish statistically significant differences) after 7 days at that O_2_ concentrations. For IH (1.5%–21% O_2_ cycles-as previously explained for 24 hours a day), we could not observe significant changes in mortality of controls after 7 days; hence we chose period of 10 days of exposure and this allowed us to distinguish differences between controls and experimental groups. Unpaired student t-tests were used to calculate significant differences in the percent survival of each P-element line as compared to the controls under each treatment.

#### b) Overexpression of Hsp70 in various tissues using specific GAL4 drivers

The UAS line for heat shock 70Bbb (yw; P{y[+t7.7] = Mae-UAS.6.11}Hsp70Bbb[UY2168]) was crossed with various GAL4 drivers which drive the expression of Hsp70 in specific tissues such as da (expresses Hsp70 in all tissues), Eaat1 (glial cells), elav-Gal4 (neurons-nervous system(CNS and PNS [50]), P{GawB}c739 (strong expression in alpha and beta lobe Kenyon cells (intrinsic neurons) of the Mushroom bodies [21]), P{GawB}DJ667 (adult muscles) and hand-Gal4(cardial cells, pericardial cells and lymph gland). The F1 progeny of these crosses (Five vials each containing ten adult males and females, n = 100) were tested under 1.5% O_2_ CH for over a period until the controls flies were dead.

### Excision of P-element lines for Hsp70Bbb and Hsp70Aa

To excise the P-element inserted in or around Hsp70Aa and Hsp70Bbb genes, virgin females of P-element lines(y[1] w[67c23]; P{y[+t7.7] w[+mC] = wHy}Hsp70Aa and y[1] w[67c23]; P{y[+t7.7] = Mae-UAS.6.11}Hsp70Bbb) were crossed with males that expressed Δ2–3 Transposase. Male or female progenies possessing both the P-element and the transposase were then individually crossed to a balancer chromosome. By this process, several precise and imprecise excision lines were established. Precise excision was confirmed by PCR amplification with Accuprime Polymerase (Invitrogen, Carlsbad, CA) followed by sequencing. The Primers used for PCR are- Hsp70Aa(fwd:GCAGATTGTTTAGCTTGTTCAGC,rev:AAACTGGTTGTTGCGGTAGG); Hsp70Bbb(fwd:GCCAAATAGAAAATTATTCAGTTCC,rev:TTTGCACTGCATATCTTCACG).

## Supporting Information

Table S1 List of Significantly altered genes for Intermittent Hypoxia.
(0.02 MB XLS)

Table S2 List of Significantly altered genes for Constant Hypoxia.
(0.06 MB XLS)

Table S3 Percentage Survival of adult flies of P-elements(or EP lines)after being exposed to 1.5% CH for 7 days.
(0.02 MB XLS)

Table S4 Percentage Survival of adult flies of P-elements (or EP lines) after being exposed to 1.5%-21% IH for 10 days.
(0.01 MB XLS)
